# Retrotransposon Insertion in the T-cell Acute Lymphocytic Leukemia 1 (*Tal1*) Gene Is Associated with Severe Renal Disease and Patchy Alopecia in Hairpatches (*Hpt*) Mice

**DOI:** 10.1371/journal.pone.0053426

**Published:** 2013-01-02

**Authors:** Vishnu Hosur, Melissa L. Cox, Lisa M. Burzenski, Rebecca L. Riding, Lynn Alley, Bonnie L. Lyons, Anoop Kavirayani, Kimberly A. Martin, Gregory A. Cox, Kenneth R. Johnson, Leonard D. Shultz

**Affiliations:** The Jackson Laboratory, Bar Harbor, Maine, United States of America; National Institute of Allergy and Infectious Diseases, United States of America

## Abstract

“Hairpatches” (*Hpt*) is a naturally occurring, autosomal semi-dominant mouse mutation. *Hpt*/*Hpt* homozygotes die in utero, while *Hpt*/+ heterozygotes exhibit progressive renal failure accompanied by patchy alopecia. This mutation is a model for the rare human disorder “glomerulonephritis with sparse hair and telangiectases" (OMIM 137940). Fine mapping localized the *Hpt* locus to a 6.7 Mb region of Chromosome 4 containing 62 known genes. Quantitative real time PCR revealed differential expression for only one gene in the interval, T-cell acute lymphocytic leukemia 1 (*Tal1*), which was highly upregulated in the kidney and skin of *Hpt*/+ mice. Southern blot analysis of *Hpt* mutant DNA indicated a new EcoRI site in the *Tal1* gene. High throughput sequencing identified an endogenous retroviral class II intracisternal A particle insertion in *Tal1* intron 4. Our data suggests that the IAP insertion in *Tal1* underlies the histopathological changes in the kidney by three weeks of age, and that glomerulosclerosis is a consequence of an initial developmental defect, progressing in severity over time. The Hairpatches mouse model allows an investigation into the effects of *Tal1,* a transcription factor characterized by complex regulation patterns, and its effects on renal disease.

## Introduction

In 1979, the Mouse Mutant Stock Center of The Jackson Laboratory identified a novel spontaneous mutation causing skin abnormalities and progressive renal disease. The mutation was named “Hairpatches” (*Hpt*) because heterozygous mice displayed characteristic abnormal hair growth patterns. An inbred strain, HPT/LeJ, was developed to reduce the phenotypic variability found in the original segregating (C57BL/6J x C3HeB/FeJ-*a/a*) hybrid background. On the HPT/LeJ strain background, *Hpt* is semi-dominant, and is lethal in the homozygous state. Postnatal, *Hpt*/+ mice exhibit renal disease similar to a progressive membranous glomerulosclerosis with striking early abnormalities of the podocytes [1].

Our previous genetic linkage analysis localized *Hpt* to a broad region on Chromosome 4, linked to pintail (*Pt*) and brown (tyrosinase-related protein 1, *Tyrp1*) [1] In the current study, *Hpt* was mapped to a 6.7 Mb region of mouse Chromosome 4, containing 62 genes. Quantitative real-time PCR (qPCR) was performed on 62 known genes in this interval. Expression of the T-cell acute lymphocytic leukemia (*Tal1*) gene (also known as stem cell leukemia, *Scl*) was upregulated in the kidney and skin of *Hpt/+* heterozygotes and was the only candidate gene having marked differential expression compared with wildtype controls. Through Sanger and high throughput DNA sequencing (HTS), we identified an intracisternal A particle (IAP) insertion in intron 4 of the murine *Tal1* locus.

*Tal1* has been studied extensively for its involvement in hematopoiesis and vasculogenesis during embryonic development and adult cell maintenance [2]. Less work has been done on *Tal1* expression in the kidney, although it has been demonstrated that *Tal1* is differentially expressed during mammalian renal development, with the highest RNA expression in mouse embryos at 17 days post-coitum (dpc) [3]. More extensive work has been done in the zebrafish model, where overexpression of *Tal1* leads to a reduction in endothelial progenitor cells destined for the kidney and skin, and also disruption of vasculogenesis in these organs [4]. This has similarities with the Hairpatches mutant, where kidney and skin are the most affected organs. In this paper we show that an IAP retroviral insertion in *Tal1* intron 4 is associated with the Hairpatches phenotype. Mechanistic aspects of the pathological effects of the IAP insertion in *Hpt*/+ mice are under investigation.

## Results

### Gross and Histopathological Changes in the Kidney and Skin of *Hpt*/+ Mice

**Figure 1:**
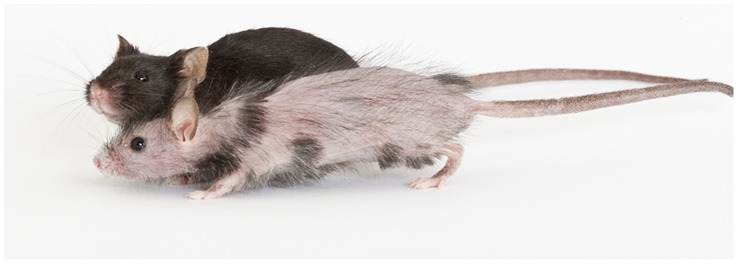
*Hpt*/+ and +/+ female mice at 19 weeks of age. The *Hpt*/+ mouse shows patchy alopecia on the trunk and head.

H*pt*/+ mice are readily identified at approximately three days of age by the patchy absence of skin pigment and at older ages by the patchy absence of hair. While a marked reduction of hair follicles is evident during embryonic development, hair follicles in adult *Hpt*/+ mice are often plugged or not erupted [1]. The patches of hair usually appear as linear transverse stripes originating at the dorsal midline. Some *Hpt*/+ mice have nearly full hair coats with only small hairless stripes, while others have more widespread alopecia interrupted with single hairs (Fig. 1). *Hpt/+* mice are occasionally born missing one kidney (unilateral renal agenesis), although the prevalence of kidney agenesis is not known because many animals are culled without necropsy during normal colony maintenance. Gross evaluation of kidneys from adult *Hpt*/+ mice shows progressive dilation of the renal pelvis, along with glomerular sclerosis and cysts visible to the naked eye. The levels of reduction in hair coat and the extent of renal pathology do not appear to be correlated.

### Progression of Glomerular Sclerosis in *Hpt*/+ Mice

**Figure 2:**
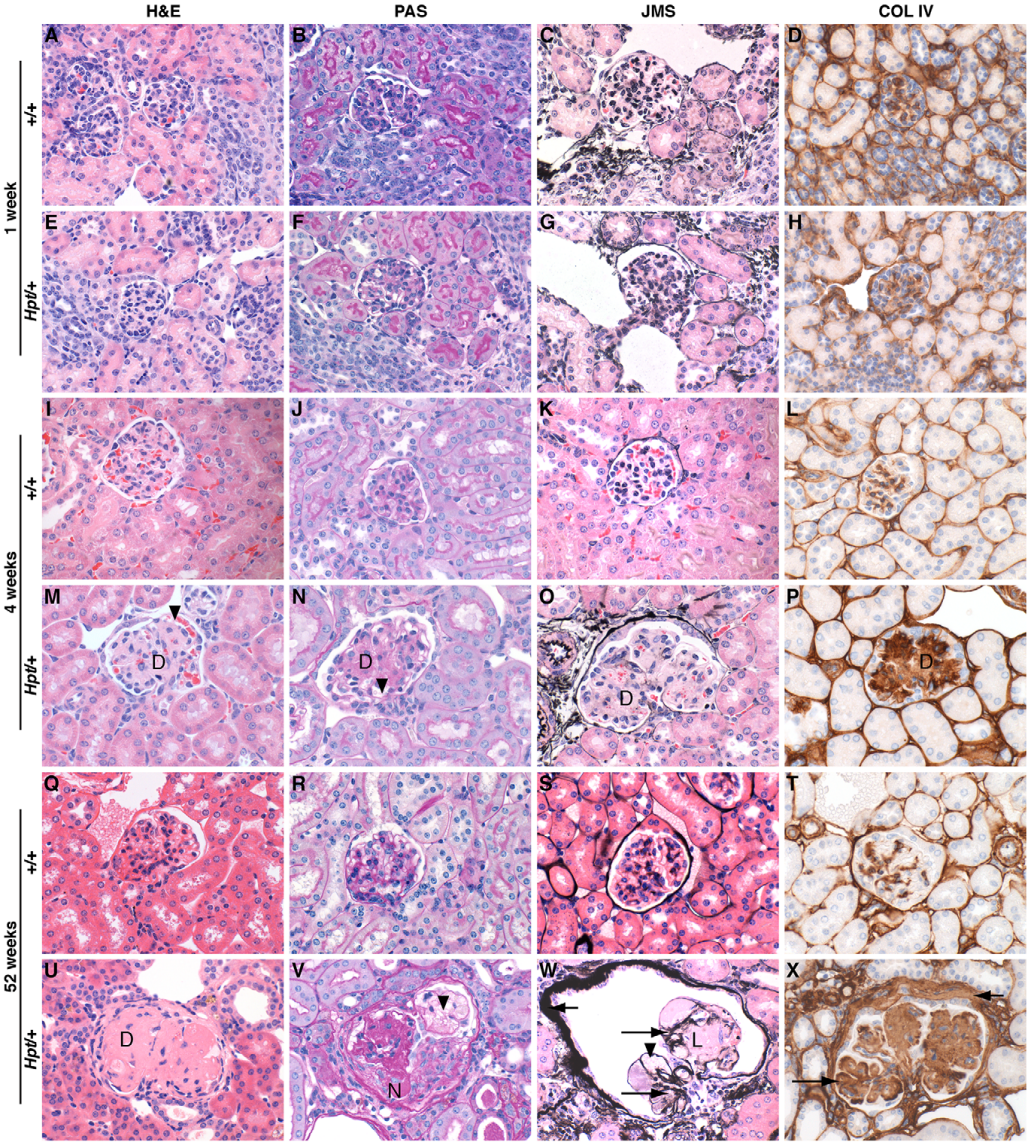
Renal histopathology of**Hpt**/+ and +/+ female mice at 1, 4, and 52 weeks of age. Representative glomeruli from *Hpt/*+ and +/+mice, at 1 week, 4 weeks, and 52 weeks of age. There are no marked histopathological changes between *Hpt*/+ and +/+ glomeruli at 1 week of age. At 4 weeks of age, *Hpt*/+ glomeruli show enlargement, diffuse mesangial matrix expansion (D) and a few dilated glomerular capillaries (arrowheads). At 12 months of age, *Hpt*/+ glomeruli show diffuse (D) and nodular (N) mesangial matrix expansion with mesangiolysis (L), capillary aneurysms (arrowheads), and thickening of glomerular (long arrows) and capsular (short arrows) basement membranes. Hematoxylin and Eosin (H&E), Periodic Acid Schiff (PAS), Jones Methenamine Silver (JMS) stains, and type IV collagen immunohistochemistry are shown; x600.

**Figure 3:**
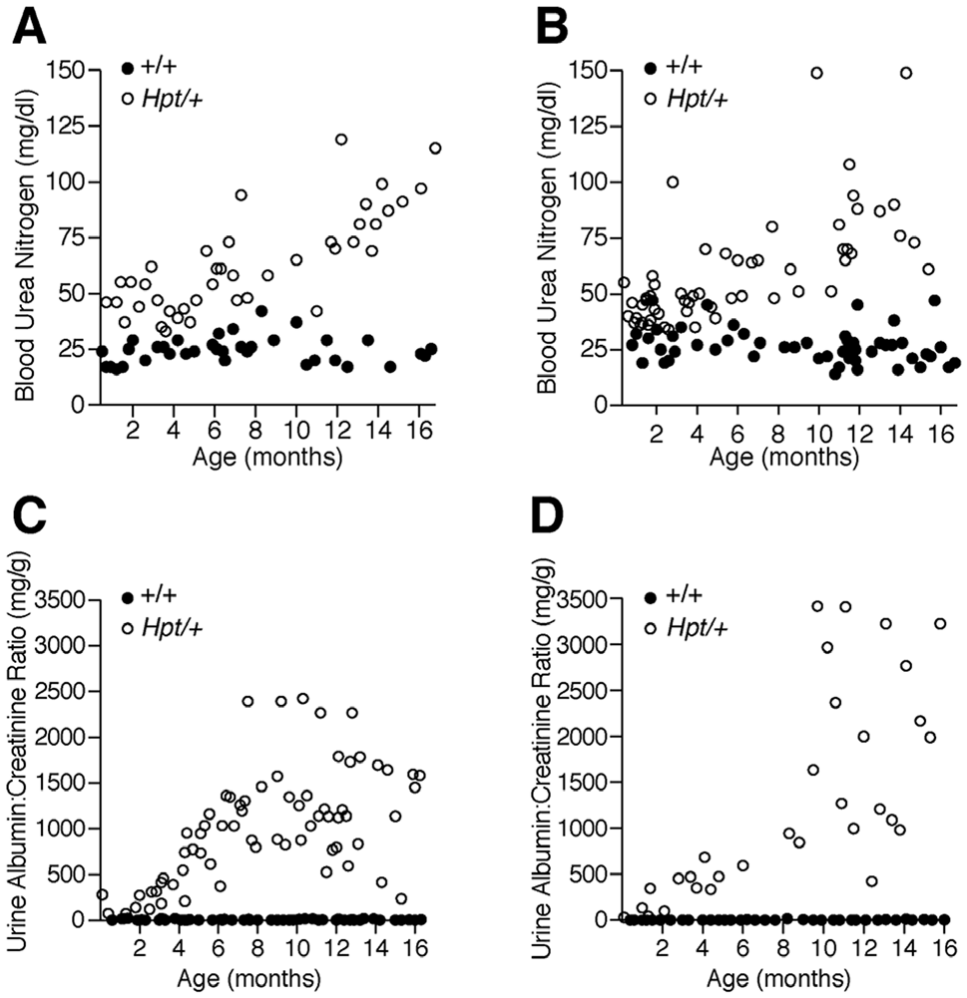
Renal function. A (male), B (female). Blood urea nitrogen (BUN) levels in individual *Hpt*/+ and +/+ mice. C (male), D (female). Urine albumin:creatinine ratio (ACR) in individual *Hpt*/+ and +/+ mice. Renal function progressively declines in the *Hpt*/+ animals. BUN values are significantly elevated (p<0.05) in *Hpt*/+ male (A) and female (B) mice compared with sex-matched +/+ controls at all time points (unpaired two-tailed T-test with Welsh’s correction). Urinary ACR is significantly elevated (P<0.05) as early as one month of age in males (C) and by three months of age in females (D) (unpaired two-tailed T-test with Welsh’s correction).

Although kidneys from *Hpt*/+ mice appear histologically normal at 1–2 weeks of age, increased glomerular mesangium is visible by 3–4 weeks, and advanced sclerosis by 6–12 months of age (Fig. 2). *Hpt*/+ mice show progressive increases in glomerular mesangial matrix accumulation, hyalinization, and marked hypertrophy with mild lymphocytic infiltration in the glomeruli and interstitium. There is extensive extracellular matrix remodeling and mesangial thickening with eosinophilic material and collagen deposits. Increases in blood urea nitrogen (BUN) levels are significant at all tested time points between 2 and 16 months of age (Figs. 3A,B). The *Hpt*/+ urinary albumin: creatinine ratio (ACR) increases steadily after three months of age (Figs. 3C,D). Heterozygotes have reduced body weight compared to wildtype animals at all time points after five months of age (Figure S1). A significant reduction in the number of circulating red blood cells was observed in *Hpt*/+ mice older than one year compared with +/+ controls. Both sexes of *Hpt/+* mice have significantly lower hemoglobin levels and hematocrit readings than +/+ mice, typical of anemia secondary to kidney disease [5]. White blood cell counts were normal (Table S1).

### The *Hpt* Mutation Maps to a 6.7 Mb Region of Chr 4

**Figure 4:**
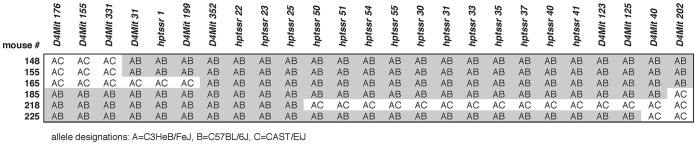
High resolution mapping of the *Hpt* mutation. Fine mapping of the *Hpt* mutation by segregation analysis of the HPT/LeJ-*Hpt*/+ x CAST/EiJ) F1 hybrid x C3HeB/FeJ cross. All of the 182 N2 offspring typed for the Chromosome 4 markers had one allele derived from C3HeB/FeJ (A) and the other allele derived from either C57BL/6J (B) or CAST/EiJ (C). Shown are the AB (gray) and AC (white) genotypes of 25 markers in the six most informative mutant N2 mice (*Hpt*/+, genotype designation AB) recombinant between *D4Mit176* and *D4Mit202*. The markers *D4Mit 352*, *hptssr22, hptssr23,* and *hptssr25* are non-recombinant with the *Hpt* mutation in all six mice, and the new flanking markers *D4Mit 199* and *hptssr50* refine the candidate gene interval to a 6.7 Mb region.

Genetic mapping and genotyping of strain-specific markers determined that the mutant *Hpt* allele originated on a C57BL/6J-derived region of Chr 4 in the original segregating hybrid background (C57BL/6J x C3HeB/FeJ-*a/a*) of the HPT/Le strain. To carry out high resolution mapping of the *Hpt* locus, F1 generation mutant offspring of HPT/Le-*Hpt*/+ males and CAST/EiJ females were crossed with C3HeB/FeJ mice. Assignments of *Hpt*/+ or +/+ genotypes of the resulting 182 N2 offspring were made on the basis of skin and kidney histology. Mice were considered as *Hpt/+* if they showed alopecia accompanied by glomerulosclerosis. Using PCR we genotyped five microsatellite markers (*D4Mit178, D4Mit27, D4Mit176, D4Mit202*, and *D4Mit203*) that differed between the CAST/EiJ strain and the C57BL/6J and C3HeB/FeJ strains in a ∼60 Mb region of Chr. 4 containing the mutation. Segregation analysis of the marker genotypes with deduced *Hpt* genotypes mapped the *Hpt* candidate region to a 26.6 Mb interval between *D4Mit176* and *D4Mit202*. DNAs of mice that exhibited recombination within this interval were then typed for 24 additional markers, including *D4Mit155, D4Mi331, D4Mi31, D4Mit199, D4Mit352, D4Mit123, D4Mit125, D4Mit40*, and 16 other informative simple sequence repeats (designated *hptssr#*). Segregation analysis of these new markers further narrowed the candidate interval to a 6.7 Mb region, between the *D4Mit199* (NCBI Build 37 position 111.55 Mb) and *hptssr50* (position 118.25 Mb) markers, containing 62 known genes (Fig. 4).

### *Tal1* Expression is Significantly Upregulated in the Affected Tissues of *Hpt*/+ Mice

**Figure 5:**
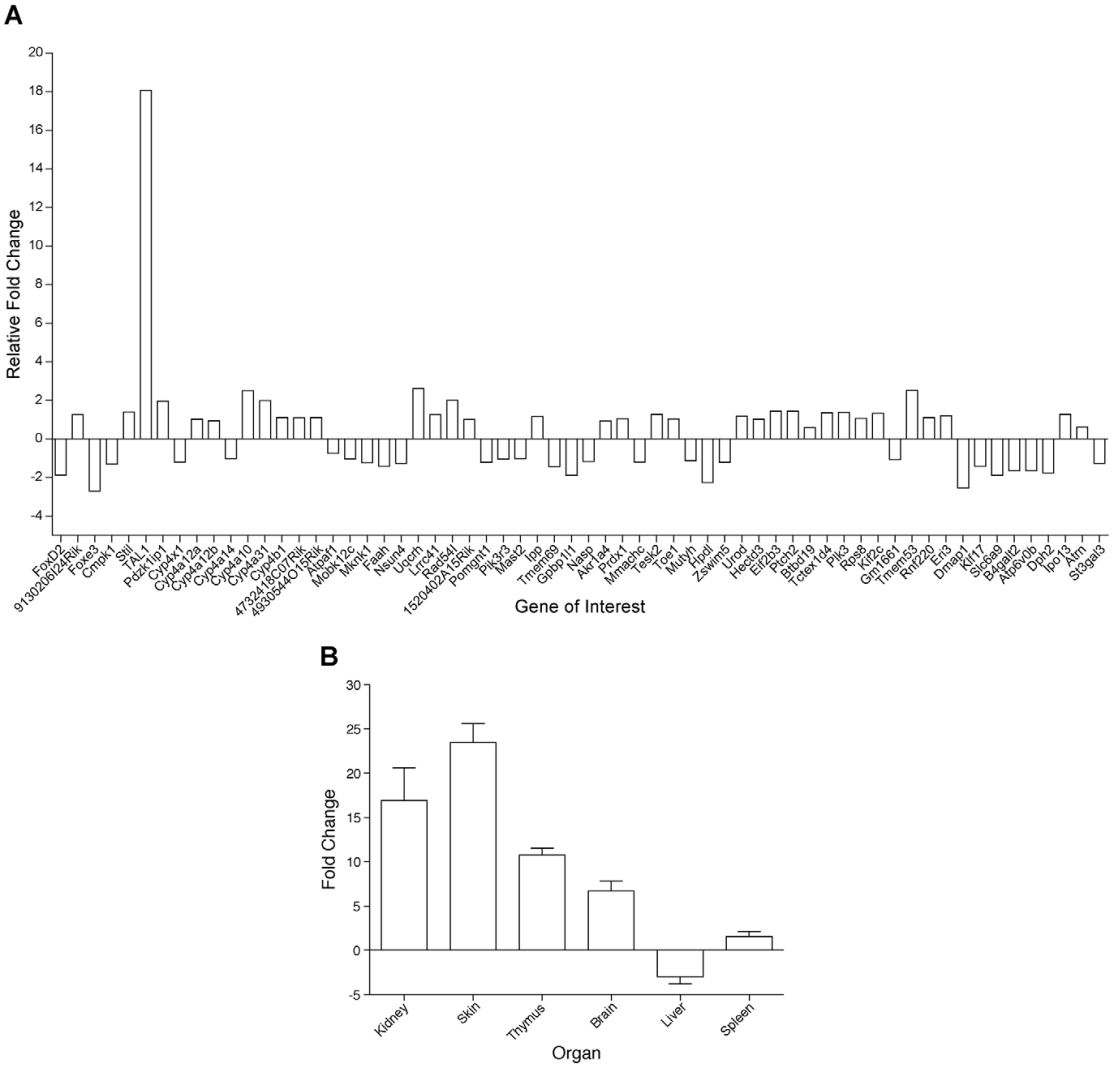
A) Sybr Green qPCR expression analysis of genes in the 6.7 Mb candidate region of Chromosome 4. Relative gene expression levels of 62 genes from the adult kidneys of *Hpt*/+ mice (1.5–15 months of age) within the candidate interval are shown (n = 3). Only *Tal1* showed a 18-fold increase, whereas no greater than 3-fold change was observed among other genes in the interval. The SybrGreen-*TAL1* Probe is located in the non-coding cDNA region of exon 5 (see Fig.7A). **B)** **Tissue-specific expression of** ***Tal1.*** Relative gene expression levels of *Tal1* in *Hpt*/+ versus +/+ animals using Sybr Green qPCR (n = 3). *Tal1* is overexpressed in the kidney, skin, thymus and brain of *Hpt*/+ mice, while there is no significant expression difference in liver or spleen. As there were no statistically significant differences in the expression values of 4 day, 14 day, 6 month, or 10–15 month *Hpt*/+ mice tested, expression values for each genotype were pooled from all age groups.

Primers were designed to carry out SybrGreen qPCR for 62 known genes in the candidate interval. cDNA was generated from random-primed total RNA from adult *Hpt*/+ and +/+ kidney tissue. Transcripts of 62 genes were successfully amplified. After normalization to the endogenous control *Hprt* gene, the expression level of the *Tal1* gene was estimated to be 18-fold higher in *Hpt*/+ kidneys compared with the +/+ controls, while no expression difference greater than 3-fold was found for any other gene in the interval (Fig. 5A). Because the SCL/*Tal1* interrupting locus (*Stil)*, located directly upstream from *Tal1*, is known to have *Tal1* regulatory regions [6], and the PDZK interacting protein 1 (*Pdzk1ip1)* gene (also known as *Map17*) shares regulatory regions and is expressed in kidney and skin [7], further samples were tested for expression of these genes, but no differences in *Stil* or *Pdzk1ip1* gene expression were found between *Hpt*/+ and +/+ mice (data not shown). To further evaluate *Tal1* expression, RNA was isolated from kidney, skin, thymus, brain, liver, and spleen of *Hpt*/+ and +/+ mice at several ages (4 days, 14 days, 6 months, 10–15 months), and SybrGreen qPCR was performed on these samples. Within the same genotype and tissue, no statistically significant *Tal1* expression differences were found between mice of different ages, so the levels of *Tal1* expression were all pooled for analysis (Fig. 5B).

### Southern Blot Analysis Indicates the *Hpt* Allele has a Novel *Eco*RI Site

**Figure 6:**
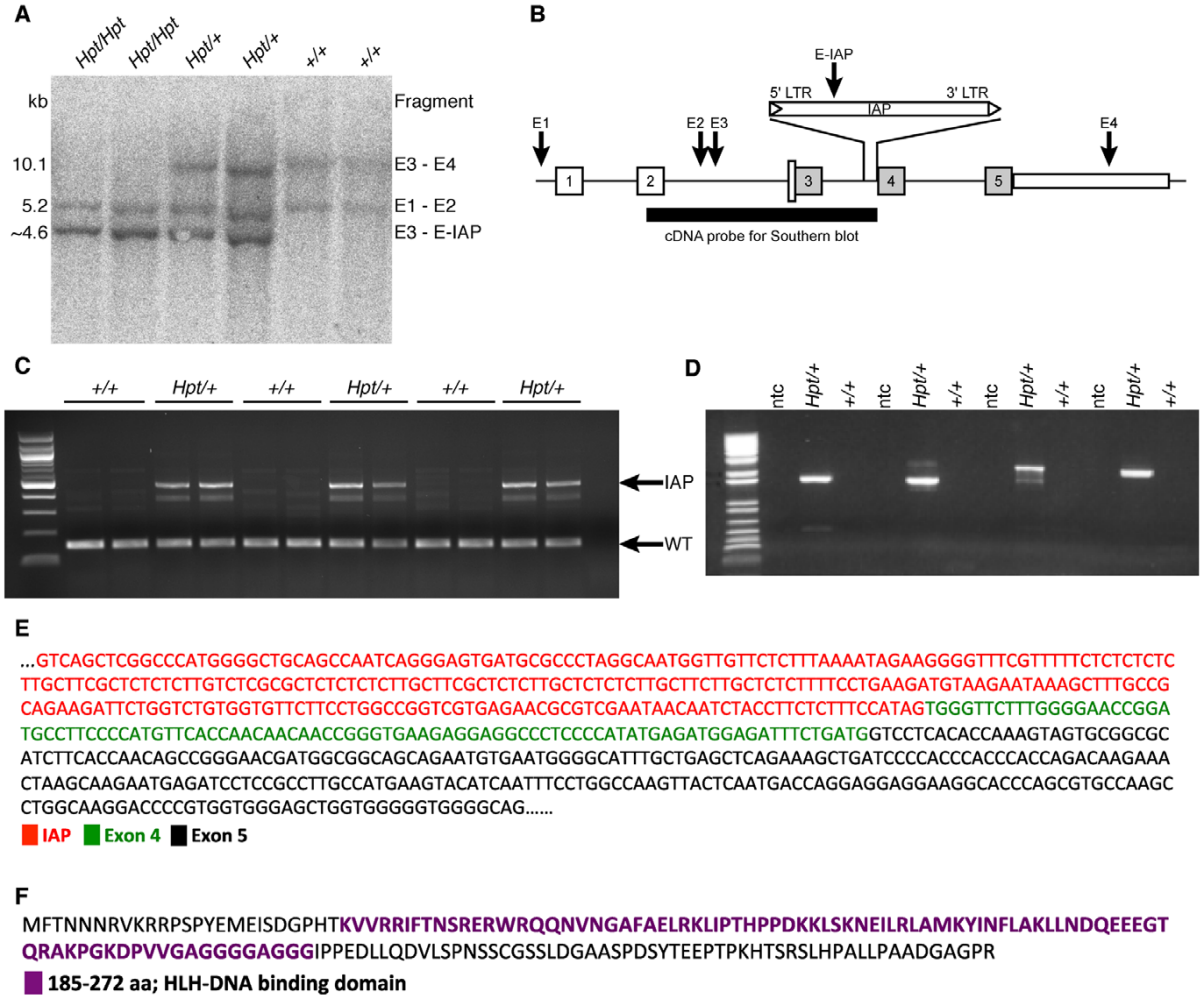
*Tal1* Southern blot and IAP insertion. There are 4 *Eco*RI sites in the murine *Tal1* gene (labeled E1–E4). Southern blot was performed on genomic DNA from 15 dpc embryos from *Hpt/Hpt*, *Hpt/+* and +/+ mice using a cDNA probe spanning exons 2–3 (A). Blotting revealed two bands in wildtype embryos: 10.1 kb (fragment E3–E4), and 5.2 kb (fragment E1–E2). *Hpt*/+ embryos show a third band, at ∼4.6 kb, representing a fragment created by the insertion of a new *Eco*RI site in intron 4. *Hpt*/*Hpt* embryos show only the 5.2 kb and 4.6 kb fragments (B). 1 µg of total RNA was reverse transcribed and PCR was performed to evaluate IAP insertion. Specific primers (Tables S3&4) were used to amplify exons 3 and 4, and IAP and exon 5 (C and D). Whereas the higher molecular weight band in *Hpt*/+ indicates IAP insertion, the lower molecular weight indicates wildtype transcript (C). Four primer sets flanking IAP and exon 5 were used on 17-day-old *Hpt*/+ and +/+ kidney cDNA to ascertain if there was a fusion product of IAP RNA with subsequent exons. ntc, no template control (D). When the product of primer set 1 (Table S4) was sequenced, the 3′ end of the IAP was spliced with exon 4 and part of exon 5 (E). Shown is the protein product that might result from using the alternative translation initiation site in exon 4, which is downstream of the IAP insertion (F).

Genomic DNA was purified from individual embryos removed at 15 dpc from females of timed matings between *Hpt*/+ mice. Genotypes of embryos (+/+, *Hpt*/+ and *Hpt/Hpt*) were determined by PCR using a combination of markers found in the mapping project to be closely linked to the *Hpt* allele. Southern blot analyses of DNA from the 15 dpc embryos of all three genotypes, as well as from C57BL/6J, and C3HeB/FeJ DNA samples, were carried out with a cDNA probe spanning exons 2–3 and intron 4 of *Tal1*. Restriction fragment length differences indicated a new *Eco*RI site in *Hpt* compared with wildtype alleles (Fig. 6A,B).

### High Throughput Sequencing Identifies an IAP Insertion in *Tal1* Intron 4

We next evaluated the entire candidate area (chr4:113,000,000–117,000,000) using high throughput sequencing. Special attention was paid to the *Tal1* locus, and all reads not properly mapped in the Chr4:114736650–114737850 region (the wild-type *Eco*RI fragment mutated in *Hpt* DNA, Fig. 6) were reassembled with Sequencher. Assembly of 1336 read sequences from mutant mice yielded 21 contigs and, in comparisons with reference C57BL/6J sequences, two of the contigs contained additional sequences matching the ends of an endogenous retroviral (ERV) class II IAP. The IAP insertion occurred in *Tal1* intron 4, between Chr 4 bp position 114,737,258 and 114,737,259. Further Sanger sequencing of this IAP element reveals that it is approximately 5 kb in length and oriented in the sense direction of the *Tal1* gene.

To demonstrate the insertion, we performed PCR analysis of *Tal1* transcripts. For this, we isolated cDNA from 2-week-old *Hpt*/+ and +/+ kidneys and performed PCR using two sets of primers (see methods); first primer set spans exons 3 and 4, whereas second set has one primer in the IAP and another in exon 5. We detected a higher molecular weight transcript only in the *Hpt*/+ kidneys (Fig. 6C,D), and when sequenced, the IAP and exon 4 were spliced together (Fig. 6E). This suggests that the IAP is inserted between exons 3 and 4, and since the insertion is in frame it can drive expression of downstream exons and produce a mutant protein product (Fig. 6F).

### The IAP Element Appears to Promote Increased Transcription of *Tal1* Exons 4 and 5

**Figure 7:**
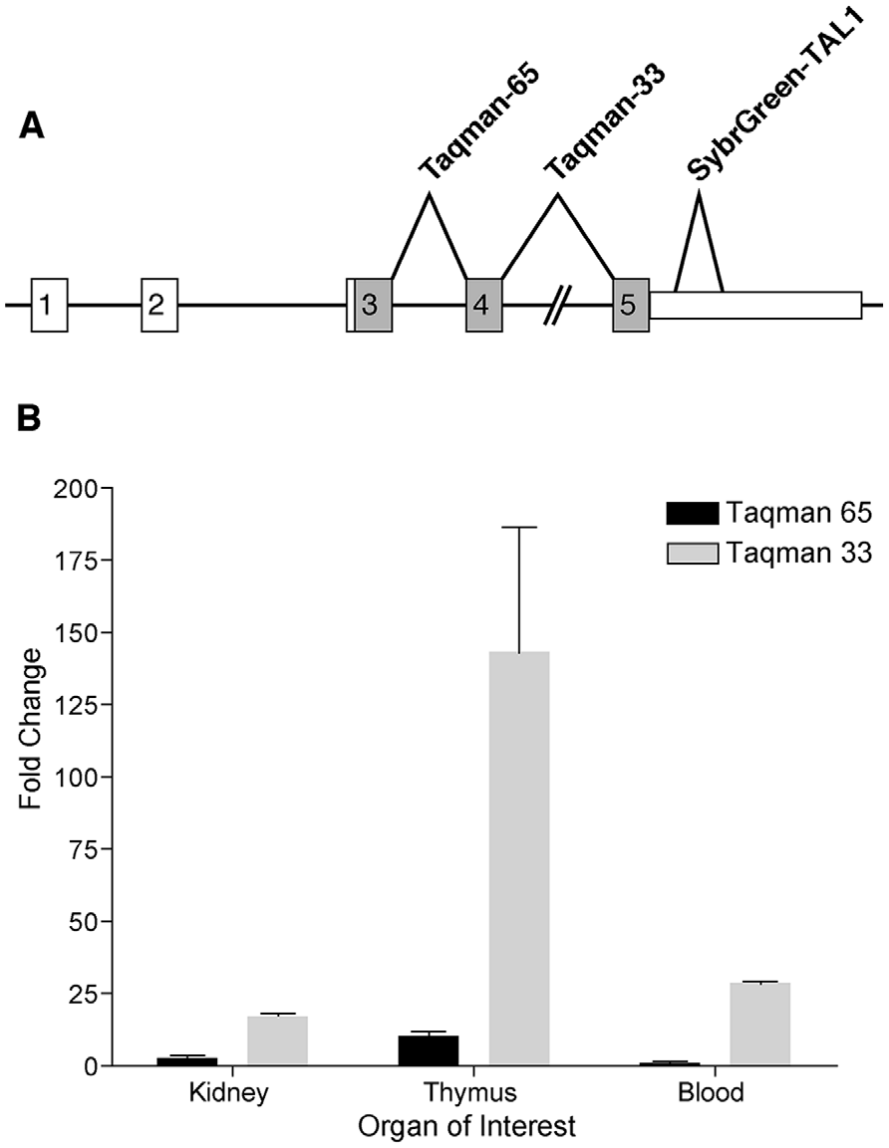
Real-time qPCR analysis of *Tal1* expression using Taqman probes. The Taqman-65 and -33 probes span exons 3 and 4, and exons 4 and 5, respectively (A). While the increased expression of *Tal1* in *Hpt*/+ mice versus +/+ controls is significant with both probe sets in all organs tested, the more 3′ Taqman-33 shows greater up-regulation than the Taqman-65 probe located more 5′(B).

Because the SybrGreen *Tal1* (SybrGreen-SB1) probe was located completely in exon 5, we used additional intron-spanning probes to ensure that genomic DNA contamination was not responsible for any of the expression differences noted in these studies. Ready-made Taqman assays were purchased from Applied Biosystems and tested on various tissues. The Taqman-65 probe spanned exons 3 and 4, while the Taqman-33 probe spanned exons 4 and 5 (Fig. 7A). Interestingly, there was a significant difference in the results obtained from the two probe sets. *Tal1* was overexpressed as previously noted in kidney and thymus and in peripheral blood leukocytes using the SybrGreen-SB1 exon 5 probe, and similar overexpression was found in all three tissues when using the Taqman-33 probe set (exons 4/5). In contrast, no difference in expression level was found in kidney or blood using the Taqman-65 probe set (exons 3/4). A moderate increase of 10.2-fold was noted in thymus with the Taqman-65 probe, but this was much smaller than the 75- to 150-fold increases detected with the other two probes (Fig. 7B). The insertion of the IAP element in intron 4 could promote transcription of exons 4 and 5, which could explain why increased expression was detected with the exon 4–5 probe (Taqman-33) and the exon 5 probe (SybrGreen-SB1) but not (or to a lesser extent) with the exon 3–4 probe (Taqman-65).

### Tal1 Protein Expression in the Kidney of *Hpt*/+ Mice

**Figure 8:**
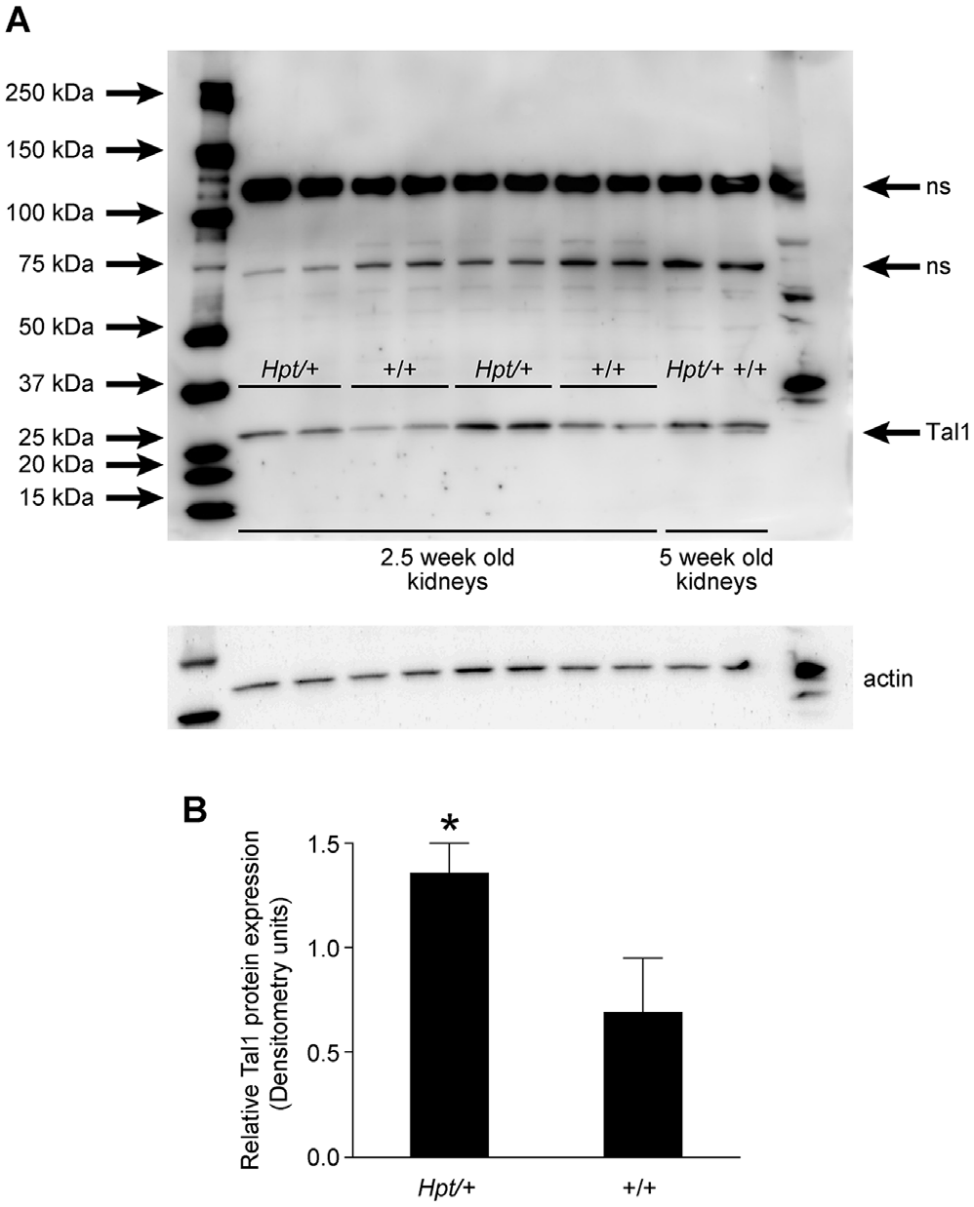
Tal1 protein levels in kidney tissues. Western blot analysis of kidney tissues from 2.5- and 5-week-old +/+ and *Hpt*/+ mice. 40 µg of protein was loaded onto each lane, and probed with c-terminal Tal1-specific antibody. A subtle but significant increase in Tal1 protein levels was observed in 2.5-week-old *Hpt*/+ mice compared with +/+ control mice, however we did not detect any shortened protein products using this antibody. Actin was used as a loading control. ns, non-specific bands (A). Densitometry quantification of Tal1 protein expression in the kidneys of 2.5-week-old *Hpt*/+ and +/+ mice (B). *p<0.05.

*Tal1* gene expression is highly regulated in the normal developing kidney. Its strongest expression is observed between embryonic days 13 and 17, but after birth the levels rapidly decrease and continue to remain low in adult kidneys [3]. Western blot analysis on kidney tissues from 2.5 and 5-week-old *Hpt*/+ and +/+ mice, using c-terminal Tal-1 specific antibodies showed a subtle but significant increase in Tal1 protein expression in 2.5-week-old *Hpt*/+ mice (Fig. 8A&B), nevertheless, our antibodies were unable to detect shortened protein products that might correspond to the mutant fusion transcript.

## Discussion

*Tal1* is a transcriptionally complex gene that is expressed throughout development, activating or repressing transcription in hematopoietic, neural, and endothelial precursors [8], [9], [10]. Human and murine *Tal1* genes share 88% identity through the coding region, although transcripts with exon 2a of the human gene have not been reported in the mouse [11]. The murine *Tal1* gene is consists of five exons (1,2, 3, 4, and 5), although only the last three are coding. The non-coding regions contain multiple regulatory regions, many of which are cell-lineage specific [11], [12], [13]. Three alternative promoters exist in both human and mouse, and at least five known tissue/cell-type specific enhancers exist both upstream and downstream of the coding region [13]. Alternate exons may be used at the 5′ end, resulting in at least six described mRNA splice variants [11].

In humans, a translocation (1;14) (p34;q11) involving *Tal1*, or a deletion 90 kb upstream of it, frequently accompanies cases of T-cell acute lymphocytic leukemia (also known as stem cell leukemia) [14]. Genetically modified mouse models have shown that concurrent overexpression of *Tal1* does not prompt tumorigenesis unless accompanied by mis-expression of the LIM domain only 1 (rhombotin 1) (*Lmo1*) gene [15]. Consistent with these findings, *Lmo1* is not mis-expressed in *Hpt/+* mice (unpublished), and *Hpt/+* mice aged up to two years do not show an increase of any cancers (unpublished data).

Targeted mutations creating null alleles of *Tal1* demonstrate lethality of homozygous embryos at 9.5–10.5 dpc due to implantation failure resulting from a complete absence of hematopoeisis [16], [17]; conditional mutants reveal abnormalities in megakaryocyte and erythrocyte development [18], [19], [20]. In zebrafish, overexpression of *Tal1* leads to a reduction in endothelial progenitor cells destined for the kidney and skin, and disruption in vasculogenesis in these organs [4]. This cell fate conversion occurs at the expense of other lineages, as there is an over-production of hemangioblasts, and a loss of one or more pronephric ducts [4]. Our data indicate that *Hpt* is a *Tal1* overexpression mutant as well, and it is interesting to note that the loss of pronephric ducts in zebrafish is consistent with the occasional finding of one-sided renal agenesis in *Hpt*/+ mice.

We determined by SNP genotyping that a deletion in *Tal1* was not responsible for the loss of the 10.1 kb wildtype band and creation of a new 4.6 kb band seen in Southern blots of *Eco*RI digested *Hpt* DNA (Fig. 6), because SNP alleles in the region of a deletion would all derive from the intact C3HeB/FeJ chromosome, rather than from both the C3HeB/FeJ and C57BL/6-Hairpatches chromosomes. There was also no indication of the creation of a new *Eco*RI site 4.6 kb downstream of the intron 3 *Eco*RI site (E3 in Fig. 6A), which strongly suggested that the mutation resulted from an insertion containing an *Eco*RI site. Although Sanger sequencing had found no insertions, high throughput sequencing yielded five unusual reads slightly 5′ to exon 4. Four reads were in the forward orientation, and one was in the reverse orientation. While one pair of each sequence mapped to the *Tal1* consensus sequence, their mate pairs mapped to several other chromosomes (two on chr 3, two on chr 7, one on chr 11). Further investigation of these reads determined that they each contained IAP long terminal repeat (LTR) sequence.

The *Hpt* mutation appears to be a dominant negative mutation, because the presence of an abnormal *Tal1* allele is associated with the Hairpatches phenotype and further the effects observed in *Hpt*/+ mice are very different from those found in *Tal1* knockout models [16], [17]. Moreover, it is likely that the fusion transcript-generated truncated protein product (Fig. 6F), which retains the helix-loop-helix (HLH) DNA binding domain, is able to bind to DNA and/or dimerize with different sets of HLH proteins than the normal Tal1 protein. Therefore, one could expect alterations in gene expression targets that normally require Tal1 or perhaps increased activation/repression of novel targets of this truncated protein. We noticed that *Hpt*/+ animals show normal development of hematopoietic lineages, but older mice (>1 year) show a significant reduction in the number of circulating red blood cells and lower hemoglobin levels, which could be a consequence of chronic renal damage [21]. The presence of the skin and kidney abnormalities in *Hpt*/+ mice at an early age indicates that the main impact of the mutation is developmental, and has lasting repercussions through the life of the animal. In mice, *Tal1* is normally expressed at its highest level at 17 dpc [3], and it is involved in renal cell proliferation, differentiation and survival [3], [22]. Previous scanning electron microscopy (SEM) studies on *Hpt*/+ kidneys observed podocyte abnormalities including swelling, disorganization, and foot process fusion [1]. We show here that histopathologic changes in the kidney by three weeks of age (the time of full kidney maturation in the mouse) suggesting that the initial “insult” to the kidney is developmental, and that progressive glomerulosclerosis is a consequence of an initial developmental defect in the kidney, progressing in severity over time.

Transposable elements are estimated to comprise approximately 40% of the mouse genome [23], and still active endogenous retroviral elements (ERVs) are estimated to cause approximately 10–12% of spontaneous mutations in the mouse genome [24]. The most common ERVs in the mouse are intracisternal a-particles (IAPs), which disrupt gene expression by introducing aberrant splicing, premature stop codons or polyadenylation sites, or by causing ectopic expression of the interrupted gene, driven by an antisense promoter in the IAP’s long terminal repeat (LTR) [25]. IAPs are also associated with epigenetic instability [25]. IAPs commonly insert in introns; a recent study identified IAP LTRs in the introns of 294 genes, and two thirds of these insertions are in an antisense orientation to the host gene. IAPs are often found in transcription factors and in genes regulating cell differentiation [26]. The *Tal1* IAP insertion appears to be a full length (approximately 5 to 7 kb) class II ERV. It is a clean insertion with a 6-bp direct repeat sequence flanking the IAP, which is in the same transcriptional orientation as the *Tal1* gene. Although *Tal1* shares common transcription domains with flanking genes *Sil* and *Pdk1zip* (*Map17*) [7], qPCR shows that the effects of the IAP insertion do not extend to these genes.

It is unclear how the *Tal1* IAP insertion causes the Hairpatches phenotype, although our results suggest that it promotes the overexpression of exons 4 and 5, which encode the DNA-binding domain of the protein and the 3'UTR. There could be three possible scenarios. First, since Tal1 protein levels were significantly higher in 2.5-week-old *Hpt*/+ kidneys, Hairpatches could be a Tal1 overexpression phenotype, which is consistent with the zebrafish model, where overexpression of *Tal1* leads to a reduction in endothelial progenitor cells destined for the kidney and skin. Second, the fusion transcript-generated truncated protein product might compete with normal Tal1 protein targets or might form heterodimers with different sets of HLH proteins and perhaps affect their activity. Third, the IAP insertion might affect the expression pattern of the *3′* Untranslated Region (UTR) of the *Tal1* gene. A wide array of functions has been attributed to the 3′ UTR regions, including the stability of mRNA and the extent of protein synthesis [27], [28]. Not surprisingly, alterations in the 3′ UTR such as overexpression or mutations are associated with pathological states [28], [29]. Elucidating the effects of overexpression of *Tal1* 3′ UTR region on skin and kidney development are underway. *Tal1* is a transcriptionally complex gene, with multiple tissue- and temporal-specific promoters, enhancers, repressors and splice variants, so determining how the gene is affected by the IAP insertion is not trivial. Studies are currently underway to fully characterize the IAP insertion in *Tal1,* determine the manner in which it causes the Hairpatches phenotype, and investigate its role in renal and skin development.

## Materials and Methods

### Mice

The inbred HPT/Le *Hpt/*+ strain was developed from a segregating hybrid stock of C57BL/6JLe and C3H/eB/FeJLe-*a/a.* Strain C3H/eB/FeJLe-*a/a* is strain C3H/eB/FeJ with the non-agouti allele from C57BL/6J backcrossed for 7 generations and then sib mated for >40 generations. The HPT/Le inbred line originated from the N3 generation of the hybrid B6C3Fe-*a/a* stock and has been mated brother x sister with one of each pair *Hpt/*+ and the other +/+ for >50 generations. The HPT/LeJ colony was maintained by male mutant (*Hpt*/+) x female wildtype (*+/+*) sib-matings. Heterozygotes are easily identifiable prior to weaning due to patchy alopecia. Timed (HPT/Le-*Hpt/*+ x HPT/Le- *Hpt*/+) matings were established to produce embryos of all three genotypes (*Hpt/Hpt, Hpt*/+ and +/+), and the dates of pregnancy were determined by the observation of vaginal plugs. Pregnant *Hpt*/+ females were euthanized between 12 and 18 days post-coitum (dpc) and embryos were collected. Genomic DNA from whole embryos was isolated using the Qiagen DNeasy Blood & Tissue Kit (Valencia, CA) per manufacturer’s instructions. Embryos were genotyped using primers for microsatellite markers JXR0418844 and JXR0418790, which were determined in the fine mapping project to distinguish polymorphisms between C57BL/6J and C3HeB/FeJ-*a/a* strains at those loci. All mice were reared on NIH 31 M diet and acidified water *ad libitum* under modified barrier conditions at The Jackson Laboratory in a 12-hr dark/12-hr light cycle. The Institutional Animal Care and Use Committee of The Jackson Laboratory approved all animal procedures.

### Genetic Mapping

A new genetic linkage cross was carried out to refine the map position of the *Hpt* mutation. HPT/Le-*Hpt*/+ mice were first mated with CAST/Ei mice. The (HPT/Le x CAST/Ei)- *Hpt*/+ F1 offspring were then crossed with C3HeB/FeJ mice to generate recombinants for mapping studies to narrow the *Hpt* candidate interval on chromosome 4. Genomic DNA was isolated from kidney tissues of 182 N2 offspring from this cross using standard protocols. Multiple Chr 4 markers were analyzed for co-segregation with the *Hpt* mutant phenotype. Microsatellite markers were typed by PCR amplification of genomic DNA with locus-specific primers, which were then distinguished by size differences on agarose gels. Mice were typed for 13 established MIT markers and new PCR primers were designed to genotype 16 additional strain-specific simple sequence repeat polymorphisms identified in the Chr. 4 region surrounding the locus. Markers that differed between CAST/Ei strain and the Hairpatches parental strain (C57BL/6JLe and C3H/eB/FeJLe-*a/a*) were used to narrow the target region (Fig. 4).

### Histology

Mice were euthanized by CO_2_ asphyxiation or cervical dislocation. Tissues were fixed in Telly’s (Tellyesniczky/Fekete) solution, 4% paraformaldehyde, or Bouin’s solution, embedded in paraffin and sectioned at 3–5 µm. Slides were stained with Mayer’s hematoxylin and eosin (H&E). Kidney sections were also stained with Periodic Acid Shiff’s (PAS), *Jones'* methenamine silver (JMS), and immunohistochemically for Collagen IV. For Collagen IV IHC, kidneys fixed in 10% NBF were subjected to antigen retrieval (Cell Conditioning 1, CC1), staining (rabbit primary [1∶500] and OmniMap anti-rabbit HRP) and chromogen detection (ChromoMap DAB) using the automated Ventana Discovery XT system (Ventana Medical Systems, Inc., AZ). Collagen IV antibody was obtained from USBiological (MA) and CC1, OmniMap anti-Rb HRP and ChromoMap DAB were obtained from Ventana.

### Blood and Urine Analysis/Clinical Assessment

Blood was collected by puncture of the superorbital vein (“submandibular bleed”) directly into EDTA-coated tubes or serum separator tubes. Hematology values were measured on the Siemens Advia 120 Hematology Analyzer (Siemens Healthcare Diagnostics, Deerfield, IL), equipped with software for mouse hematological analysis. Clinical pathology tests using serum were performed on the Beckman DXC Delta Clinical System (Beckman Coulter Inc., Fullerton, CA). The following methods and Beckman test kits were used: blood urea nitrogen (BUN) – enzymatic – kit # 442750; calcium – arsenazo method –kit # 442755; Alkaline phosphatase - kinetic rate – kit # 442670; total protein – Biuret –kit # 442740; iron – FerroZine – kit # 467910. Enzymatic creatinine was measured with Diazyme kit # Dz072B-K (Diazyme, Poway, CA) and albumin was measured using the colorimetric BCG method Stanbio kit # 0285-250 (Stanbio Laboratory, Boerne, TX).

### Quantitative Real-time RT-PCR

RNA was extracted from kidney, skin, thymus, brain, liver, and spleen using the RNAqueous-4PCR (Ambion, Austin, TX) system per manufacturer’s instructions, including the optional DNase treatment. RNA was extracted from whole blood using the Mouse RiboPure-Blood RNA Isolation Kit (Ambion). A Nanodrop ND-1000 UV spectrophotometer (ThermoScientific Nanodrop Products, Wilmington, DE) was used to determine RNA concentration. RNA quality was assessed by capillary electrophoresis using an Agilent Bioanalyzer 2100 (Palo Alto, CA), from which an RNA Integrity Number (RIN) was calculated by the Agilent 2100 Expert software. RNA samples with a RIN of eight or above were used in the qPCR. Enhanced Avian Reverse Transcriptase (Sigma Aldrich, St Louis, MO) was used to reverse transcribe 1 µg total RNA using random primers per manufacturer’s instructions.

Gene-specific primers were designed for SybrGreen q-RTPCR for 62 known genes within the 6.7 Mb candidate interval on Chr. 4 (Table S2). SYBR-Green PCR Master Mix (Applied Biosystems, Foster City, CA) was used per manufacturer’s instructions with 10 ng kidney cDNA. To verify *Tal1* up-regulation as shown by SYBRGreen qPCR, two *Tal1* Taqman ready-made assays (Mm01187033_m1, Mm00441665_m1) were purchased from Applied Biosystems (Foster City, CA), along with an endogenous control, hypoxanthine guanine phosphoribosyl transferase 1 (HPRT) (Mm03024075_m1). cDNA (50 ng) was subjected to qPCR per manufacturer’s instructions.

For SYBRGreen and Taqman assays, each cDNA sample was run in triplicate on an Applied Biosystems 7500 Real-Time PCR System on at least two different occasions, and specificity of the amplification was verified by agarose gel electrophoresis. Using the ABI Prism SDS software, triplicate determinations were averaged and mean Ct calculated from the two replicates from one plate. Individual expression values were normalized by comparison to HPRT. Finally, a mean Ct was calculated from reactions performed at different times. Failed reactions were not included. The final control mean Ct was subtracted from each final sample mean Ct.

### Southern Blot

Genomic DNA samples (2.5 µg) from two 14.5 dpc embryos of each genotype (*Hpt*/*Hpt*, *Hpt*/+, and +/+) and from control C657BL/6J and C3HeB/FeJ-*a/a* wildtype DNA (purchased from the JAX Mice DNA Repository) were digested with 10 U *Eco*RI, *Msp*I, *Pst*I, *Taq*I, or *Xba*I restriction enzymes (Promega, Madison, WI) for 12 hours at 37°C or 65°C. After resolution on a 0.8% agarose gel, DNAs were vacuum blotted to a positively charged nylon membrane using the VacuGeneXL vacuum blotting system (Amersham, GE Biosciences, Piscataway, NJ) with standard depurination, denaturation, and neutralization solutions. DNAs were crosslinked to the membrane using the UV Stratalinker 2400 (Stratagene, La Jolla, CA). A *Tal1* cDNA probe, spanning exons 2–3 was amplified using the Advantage GC2 Polymerase kit (Clontech) with the Roche PCR DIG probe Synthesis Mix (Roche Applied Science, Indianapolis, IN) containing DIG-labeled dUTP. We performed hybridization by adding denatured DIG-labeled probe to the DIG Easy Hyb solution (Roche) and incubating per manufacturer’s instructions. Post-hybridization high and low stringency buffers were prepared according to standard protocols, and washing and blocking were performed using the components from the DIG Wash and Block Buffer Set (Roche). Ready to use CDP-*Star* (Roche) was employed to generate the luminescent signal, which was detected on autoradiographic film (Eastman Kodak Co., Rochester, NY) after 15–30 minute exposures. A novel band in EcoRI-digested *Hpt*/+ and *Hpt/Hpt* embryos was found and the membrane was hybridized with a P^32^-labeled *Tal1*-cDNA probe using previously described standard protocols [30].

### Sequencing of *Tal1*

*Tal1* primers were designed using a combination of the NCBI Primer-BLAST and NetPrimer (PremierBiosoft, Palo Alto, CA) tools. The Advantage-GC2 PCR kit (Clontech, Mountain View, CA) was used per manufacturer’s instructions, including 25 ng/µl genomic DNA. Products were resolved on 1.2% agarose gels (Low-EEO, Fisher BioReagents, Fairlawn, NJ). Bands of interest were excised and gel extracted using the QIAquick Gel Extraction kit (Qiagen) and eluted in 30 µl double distilled water. If gel extraction was not necessary to separate multiple bands, PCR products were directly cleaned using ExoSAP-It (USB, Cleveland, OH). DNA samples were quantitated using the Nanodrop ND-1000 UV spectrophotometer (Nanodrop Technologies, Wilmington, DE). Sequencing reactions with gene-specific primers were carried out using the BigDye Terminatory Cycle Sequencing chemistry and resolved on the AB3703*xl* (Applied Biosystems Life Technologies, Carlsbad, CA). Twenty-one kb of genomic DNA (including upstream conserved elements, 5′ and 3′ *Tal1* UTR, and non-coding exons) was sequenced from both strands of six homozygous mutant (*Hpt/Hpt*) and four wildtype (+/+) embryos. Sequencher 4.9 (Gene Codes, Ann Arbor, MI) was used to assemble DNA sequences.

### High Throughput Sequencing of Chr 4 Region of Interest

As no mutations were found by direct sequencing of *Tal1*, a SureSelect DNA Capture Array 1 M (Agilent Technologies Inc., Santa Clara, CA) was designed using eArray v7.0 to investigate *Mus musculus* chr4:113,000,000–117,000,000. Using an average probe spacing of 2 bp with masked repeats skipped, 822,791 60-mer probes were designed for the region of interest. A paired-end HTS library size selected to 250–350 bp, was made following the Hodges protocol (Hodges et al., 2009) from one *Hpt*/+ mouse. The library was hybridized to the Agilent SureSelect 1 M capture array, and the Seqence Captured library was run on the Illumina CAIIx (Illumina, San Diego, CA) and paired-end sequenced at 75 base read length. All reads not properly mapped in chr4:114736650–114737850 were reassembled with Sequencher, and resulting contigs were compared to C57BL/6J sequences.

### Western Blot

Kidney tissues were collected from *Hpt*/+ and +/+ animals at two different ages (2.5 weeks and 5 weeks). Total cellular protein lysates were prepared in Tris-buffered saline with 1% Igepal (Sigma-Aldrich) and protein inhibitors (Roche Diagnostics, Indianapolis, IN) on ice. Qubit Fluorometer analyzed total protein levels. Equivalent amount of protein was run on a 4–12% Tris-glycine Lonza PAGEr Gold Precast gel (Lonza, Allendale, NJ) in a Cambrex PAGEr Minigel Chamber (East Rutherford, NJ). Protein was transferred using iBLOT (Invitrogen,), and blocked with 5% non-fat dry milk in 1× TBS and 0.05% Tween 20. Blots were probed with Tal1-specific rabbit polyclonal antibody (Aviva System Biology, San Diego, CA). A protein loading control was performed by stripping (62.5 mM Tris-HCl [pH 6.7], 100 mM 2-mercaptomethanol, and 2% sodium dodecyl sulfate) blots at 50°C and re-probing with actin (1∶1000, Cell Signaling Technology, MA). Detection of antibodies was performed using HRP-conjugated anti-rabbit (1∶2000) secondary antibody and the West Pico (Pierce, USA) chemiluminescent system, followed by visualization with a CCD camera.

## Supporting Information

Figure S1 **Body weights of** ***Hpt/*** **+ and +/+ mice.** Body weights in Male (A) and female (B) *Hpt*+ and +/+ mice. *Hpt*/+ mice have reduced body weights after five months of age.
(TIF)

Table S1 **Hematological changes in aged** ***Hpt*** **/+ mice.**
(DOC)

Table S2 ***Tal1*** **sequencing, mapping, Southern blot, genotyping, and qPCR primer sets.**
(DOC)

Table S3 **Primers used in** **Figure 6C** **.**
(DOC)

Table S4 **Primers used in** **Figure 6D** **.**
(DOC)
