# Supplementary figures and images for: Retrotransposon Insertion in the T-cell Acute Lymphocytic Leukemia 1 (Tal1) Gene Is Associated with Severe Renal Disease and Patchy Alopecia in Hairpatches (Hpt) Mice
Source: PLoS One. 2013 Jan 2;8(1):e53426. doi: 10.1371/journal.pone.0053426 (PMC3534690; doi:10.1371/journal.pone.0053426)

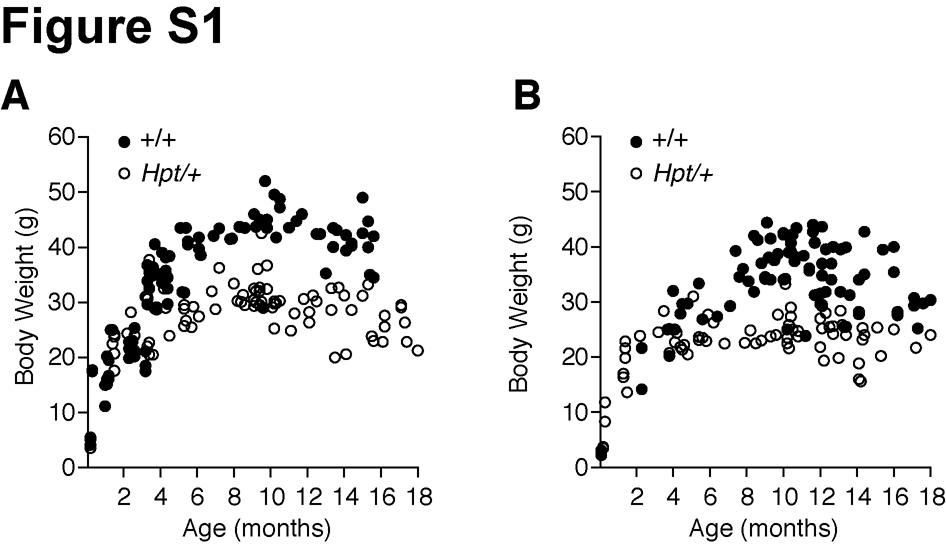

Supplement: Figure S1 — Body weights of Hpt/ + and +/+ mice. Body weights in Male (A) and female (B) Hpt+ and +/+ mice. Hpt/+ mice have reduced body weights after five months of age. (TIF) [file pone.0053426.s001.tif]
