# Supplementary material for: Retrotransposon Insertion in the T-cell Acute Lymphocytic Leukemia 1 (Tal1) Gene Is Associated with Severe Renal Disease and Patchy Alopecia in Hairpatches (Hpt) Mice
Source: PLoS One. 2013 Jan 2;8(1):e53426. doi: 10.1371/journal.pone.0053426 (PMC3534690; doi:10.1371/journal.pone.0053426)
Supplement: Table S1 — Hematological changes in aged Hpt /+ mice. (DOC) [file pone.0053426.s002.doc]

**Table S1. Hematological changes in aged *Hpt*/+ mice**

| Genotypes of mice | WBC  (x10^3^/uL) | RBC  (x10^6^/uL) | HGB  (g/dL) | HCT  (%) |
| --- | --- | --- | --- | --- |
| Combined Sexes +/+ (24) | 7.4±0.7 | 10.6±0.1 | 14.4±0.2 | 48.9±0.7 |
| Combined Sexes *Hpt*/+ (18) | 7.9±0.7 | 9.8±0.4 | 11.5±0.3 | 40.3±0.9 |
| P value | 0.5815 | 0.0239 | <0.0001 | <0.0001 |
