# Supplementary material for: Retrotransposon Insertion in the T-cell Acute Lymphocytic Leukemia 1 (Tal1) Gene Is Associated with Severe Renal Disease and Patchy Alopecia in Hairpatches (Hpt) Mice
Source: PLoS One. 2013 Jan 2;8(1):e53426. doi: 10.1371/journal.pone.0053426 (PMC3534690; doi:10.1371/journal.pone.0053426)
Supplement: Table S2 — Tal1 sequencing, mapping, Southern blot, genotyping, and qPCR primer sets. (DOC) [file pone.0053426.s003.doc]

**Table S2**

*Tal1* sequencing, mapping, Southern blot, genotyping, and qPCR primer sets

| **Primer Name** | **Forward** | **Reverse** | **Fragment Size** |
| --- | --- | --- | --- |
| TAL1-Sybr Green | CTGTGTCAATCCAGGTGGTG | GATGGGGCCTTTGGATATTT | 183 |
| TAL1-DNA-40 | GATGACCATCGGCAGCTATT | TGGAAAGGGCTTTGCTCTAA | 480 |
| TAL1-V | GAGAGCCCGCTGCCTTCTGC | CGCCCTCCAGAGTCCAGGGT | 919 |
| TAL1-I | TGAAGTGTTTAGGTCTGGCACGATACAA | TCAGGAGAGGGAATGGGGTAAGGCT | 751 |
| TAL1-DNA-27 | CAGAGTGCTGGCAGACCGAA | TCCTGGGTGTGAAATGGCCT | 571 |
| TAL1-W | GGTGGAGGAGGGCTGGCATT | TTGGTGTGGGGAGCTGGGAG | 618 |
| TAL1-DNA-67 | ACATCCTGGATTCTTCGATTTTCAT | GTATGTGAGCTAACTGGGTGTGCTT | 910 |
| TAL1-DNA-41 | CACTTAGCCTTCACCTCCCA | GGGTGGAAGAATTGAGGGTT | 652 |
| TAL1-Y | TCCGATCTTACATAGGACAGCAAGCC | CCATAGACCCCACTAAGCATCCCAA | 424 |
| TAL1-DNA-29 | CCTGAAATCTAGGAGGGGCAGAA | GCAGGCAGGCAGATCCTTTG | 460 |
| TAL1-Z | TATGGCAACCAGACAAATGCCTATGA | AGGAACCCAATGGGCTGAGGAG | 1044 |
| TAL1-DNA-30 | ATCTGCCTGCCTGCCTCAAT | GAGGCAGAGGGAGGTGGACA | 690 |
| TAL1-DNA-20 | CATGGTCTCACTCACATAGCCC | CCAAGGACTCAATTCTCAGCAC | 649 |
| TAL1-DNA-31 | CACCAATGGGGAGAAGTTGGA | GGGATTCCCGCTCCCTACAA | 857 |
| TAL1-DNA-44 | CCAGTTTTAGAGCGGTCAGG | GAAGGAAGAGAGTCTCCCCG | 759 |
| TAL1-DNA-66 | GTTTCCTCCGTCTTTCCCCAT | ACTGACCAGAAATAGGCGAAACC | 1004 |
| TAL1-DNA-2 | ATTGCCCGTCCGTTTCCTCCGTCTTTC | ATTGTCTTTCCCAGCCTCTCCCAACCACC | 1061 |
| TAL1-DNA-32 | GTCTGTGCCTTGGTGTTGCG | GGCTTTCAATTCAGTGCGGG | 754 |
| TAL1-DNA-3 | GGCTGTCTGTGCCTTGGTGTTGC | GATTTCGTTTGTTTTATTTTCGCCTCCTTT | 915 |
| TAL1-T | GCCTCCAGCAGGGCTCTTT | TGTGCTTCCCGAGACCCAG | 558 |
| TAL1-DNA-5 | AGCTCACCAGTTCTACCCTTTAGCTTCTTTG | ATTTCACCATTTCTTCCACCACCTTTGC | 611 |
| TAL1-DNA-65 | ATTAGCCTCAAGTAACAACGGGAAC | ATACTGTTTCGCAGCGATAAACAAA | 349 |
| TAL1-DNA-33 | CTTGGAAGTGGCGTCGGTCT | AGGTAGGACCCCGAAGCTGG | 955 |
| TAL1-DNA-58.2 | CTAAGTAACTGCTGGTCTCTCAGCG | CAACCCAGAATACTGTTGTTTCCAC | 206 |
| TAL1-M2 | GCACACGAGGTAATTCCCAGTCATTG | AGCCTCAACCTCATCTTCACCTTCCAGT | 989 |
| TAL1-U | GAAGATGAGGTTGAGGCTGTAACCTGG | GGATTAGCCCAGTCAGGAAACAACAAA | 712 |
| TAL1-DNA-21 | GACAGCGTCTCTGGCTTGTGTATTT | ATAAATCACATTGCTGCTCCCAAGA | 739 |
| TAL1-DNA-22 | CAGCTTGTGTGTAACAAGTGTTGGG | CTCCAGTCGTGGATCTCACTGTTTT | 972 |
| TAL1-DNA-35 | AAACAACTCACAAGTATCATCCAA | ACCCTTCACACTCCACCCCA | 664 |
| TAL1-DNA-59 | GGGTCCATGTGAGACAGTAAAACAG | TCCCCTCCTGATGGAGAAAATATAA | 403 |
| TAL1-DNA-23 | GCAAGAACTCTCAAACTGATGGGAA | AAAGCAGCCTACACTAAGCAAACC | 674 |
| TAL1-DNA-36 | CCCAGTGAGATCCGAGTTCTGTG | CGGGGAATCTGTCCAGTGCT | 483 |
| TAL1-DNA-47 | AACGGTGGGTTTGCTTAGTG | GTCCTGTCCCTCTAGTTGCG | 659 |
| TAL1-DNA-46 | AGATCGGGAGAGGGTTCCTA | TGGAATCTTAAGCCACCACC | 966 |
| TAL1-N | AGGTTTCAGTGCGTGATCTCCTCT | TTCTACACAACTGTCGTCCCCTG | 673 |
| TAL1-DNA-48 | CGCAACTAGAGGGACAGGAC | ACAGAAGCGAAGAGGGTGAA | 1348 |
| TAL1-DNA-49 | CAGACTGTCAGGAAGGACCG | CACATTGTCCCCTCAGACCT | 561 |
| TAL1-DNA-24 | GCGTGGTTGGTCTCCTTGCT | AATTCGCCTCCAGGGTGAAGAC | 822 |
| TAL1-Q | CAATGTGAGACCCTTTGTGGTGTGGGAGTT | ATCCTTGACAAAGTGCAGGGAGCAAGCCTA | 706 |
| TAL1-DNA-60 | CTTGTCTCTGAGCAAAGGTCTACCA | CTCCTTAGATGGATTCTCCCTGTGT | 270 |
| TAL1-P | TACTTCAGCACAGGAATACGCC | TCAAGATGCAGTTGAGGGACTTT | 619 |
| TAL1-DNA-50 | ACTGGCACTTAAGCCACACC | GGAAGAGTGATGGAGGGGTC | 243 |
| TAL1-DNA-37 | GGATGCCTTCCCCATGTTCA | GGGCTGTTGTGGTTCCTGGT | 874 |
| TAL1-DNA-61 | CACGGGTAGTTCTCACTTTAGGCTT | AACAGCACTTAGAATCCCCACATTT | 453 |
| TAL1-DNA-62 | GAATTTCTGACTATACTCGTGCCCA | GGCTCTAAATTTCCACTCATCCAAG | 234 |
| TAL1-DNA-25 | CATGTCCCCAGTGCCAATGA | CAGGCTGTGGATGAGTCCCA | 674 |
| TAL1-S | CTTCTCCAGCTCCTGATAGGT | ATAGACAACCATACACAGCCAT | 1260 |
| TAL1-R | TTGAACTTCCTGAAGGTTGTCTT | CACATGCTAGGCTGGGATTAA | 1259 |
| TAL1-DNA-51 | AGCAAAGGCTGAAGCAGAAG | CTTGGCCAGGAAATTGATGT | 869 |
| TAL1-SB1 | GTCCTCACACCAAAGTAGTGCGG | TGGCTCCTCTGTGTAACTGTCCG | 377 |
| TAL1-SB2 | GAATGAGATCCTCCGCCTTGCCATGAAGTACAT | TTGGGCTTGGGAAAGGGAGAAGACCGTG |  |
| TAL1-SB3 | CTTAACATGAAACGAGTGCCCCCTCTAAAACT | ACAAAGAATCACAATGCCTTAGTAGCTGACCACA | 538 |
| TAL1-DNA-63 | ATGGGACAATGTGGTCAGCTACTAA | CAACAAAAGTGTGATCCCTCTCATA | 231 |
| TAL1-DNA-26 | GCTGGTTCTTTCCCATTGCG | AGCCTTCGGCAGAGGGTCAT | 985 |
| TAL1-DNA-55 | TTGGGTCCGTAAAGACTGCT | GTACGGCTAGACCCACCAAA | 697 |
| TAL1-DNA-26 | GCTGGTTCTTTCCCATTGCG | AGCCTTCGGCAGAGGGTCAT | 985 |
| TAL1-DNA-56 | CAGAAACAAAAACCAAGGGC | TCTAGGCTGTCTAGGGCTGC | 626 |
| TAL1-DNA-39 | TAGCACGCCATGTCTGTGGG | CCCTGGGACAAAACTGTCTCTGA | 1263 |
| TAL1-E&H | GAAGACCCTGAATTCTGTTCTCATA | AATGAAAAAGATGGCACCCTA |  |
| TAL1-H | CCAGTGTGGTGATGATAGTGTGT | AATGAAAAAGATGGCACCCTA |  |
| TAL1-DNA-A | GCAAGCTAAGTAACTGCTGGTCTCTAG | CCAAAGAACCCACTGCCTAGTGA |  |
