# Supplementary material for: Retrotransposon Insertion in the T-cell Acute Lymphocytic Leukemia 1 (Tal1) Gene Is Associated with Severe Renal Disease and Patchy Alopecia in Hairpatches (Hpt) Mice
Source: PLoS One. 2013 Jan 2;8(1):e53426. doi: 10.1371/journal.pone.0053426 (PMC3534690; doi:10.1371/journal.pone.0053426)
Supplement: Table S3 — Primers used in Figure 6C . (DOC) [file pone.0053426.s004.doc]

**Table S3**

Primers used in Fig. 6C

| Tal1 insert F | CGCTGCTCTATAGCCTTAGCC |
| --- | --- |
| Tal1 insert R | TGGTGTGAGGACCATCAGAA |
