# Supplementary material for: Retrotransposon Insertion in the T-cell Acute Lymphocytic Leukemia 1 (Tal1) Gene Is Associated with Severe Renal Disease and Patchy Alopecia in Hairpatches (Hpt) Mice
Source: PLoS One. 2013 Jan 2;8(1):e53426. doi: 10.1371/journal.pone.0053426 (PMC3534690; doi:10.1371/journal.pone.0053426)
Supplement: Table S4 — Primers used in Figure 6D . (DOC) [file pone.0053426.s005.doc]

**Table S4**

Primers used in Fig. 6D

| Tal1 IAP Set 1 Forward Primer | ATT AAC CCA CGC GGT ATT CGA CCT |
| --- | --- |
| Tal1 Ex5 coding Set 1 Reverse | TTG GGT GTT GGC TCC TCT GTG TAA |
| Tal1 IAP Set 1 Forward Primer | ATT AAC CCA CGC GGT ATT CGA CCT |
| Tal1 Ex5 coding Set 5 Reverse | TGT TGG CTC CTC TGT GTA ACT GTC |
| Tal1 IAP Set 2 Forward Primer | CAA ACC TTG GCA GCC GCA TCT AAT |
| Tal1 Ex5 coding Set 1 Reverse | TTG GGT GTT GGC TCC TCT GTG TAA |
| Tal1 IAP Set 2 Forward Primer | CAA ACC TTG GCA GCC GCA TCT AAT |
| Tal1 Ex5 coding Set 5 Reverse | TGT TGG CTC CTC TGT GTA ACT GTC |
